# Alzheimer and vascular brain disease: Senile dementia

**DOI:** 10.1590/1980-57642015DN92000013

**Published:** 2015

**Authors:** Eliasz Engelhardt, Lea T. Grinberg

**Affiliations:** 1 Full Professor (retired), Cognitive and Behavioral Neurology Unit - Institute of Neurology /Institute of Psychiatry - Federal University of Rio de Janeiro (UFRJ), Rio de Janeiro RJ, Brazil.; 2 Assistant Professor, Department of Neurology and Pathology, University of California, San Francisco, San Francisco, USA.; 3 Professora-doutora, Departamento de Patologia, Faculdade de Medicina da Universidade de Sao Paulo, Sao Paulo SP, Brazil.

**Keywords:** Alzheimer, brain vascular disease, arteriosclerosis, Senile dementia

## Abstract

Alois Alzheimer is best known for his description of a novel disease, subsequently named after him. However, his wide range of interests also included vascular brain diseases. He described Senile dementia, a highly heterogeneous condition, and was able not only to distinguish it from syphilitic brain disease, but also to discriminate two clinicopathological subtypes, that may be labeled a "arteriosclerotic subtype", comparable to the present clinicopathological continuum of "Vascular cognitive impairment", and another as a "neurodegenerative subtype", characterized by primary [cortical] ganglion cell [nerve cells] degeneration, possibly foreshadowing a peculiar presenile disease that he was to describe some years later and would carry his name. He also considered the possibility of a senile presentation of this disease subtype, which was described by Oskar Fischer a short time later. Considering the clinicopathological overlapping features of the "arteriosclerotic subtype" of Senile dementia with Arteriosclerotic atrophy of the brain, it might be possible to consider that both represent a single condition.

## Abstract (pt)

Alois Alzheimer é conhecido principalmente por sua descrição de uma nova doença, logo designado com seu nome. Entretanto, sua ampla gama de interesses, compreendia também doenças vasculares cerebrais. Descreveu a Demência senil, uma condição muito heterogênea, e foi capaz, não somente distingui-la da doença cerebral sifilítica, como também separar dois subtipos clinicopatológicos que podem ser rotulados como um "subtipo arteriosclerótico", que pode ser relacionado ao atual *continuum* clinicopatológico do "Comprometimento cognitivo vascular", e um "subtipo neurodegenerativo", caracterizado por degeneração [cortical] primária de células ganglionares [células nervosas], possivelmente prenunciando uma doença peculiar pré-senil, que ele descreveu alguns anos depois e que portaria seu nome. Considerou a possibilidade de uma apresentação senil deste subtipo da doença, que seria descrita por Oskar Fischer pouco tempo depois. Levando em conta os aspectos de sobreposição clinicopatológica do "subtipo arteriosclerótico" da Demência senil com a Atrofia arteriosclerótica do cérebro, poderia ser possível considerar que ambas representariam uma só condição.

## INTRODUCTION

Aloysius [Alois] Alzheimer (1864-1915), was a German psychiatrist and neuropathologist, best known for his description of "neurofibrillary tangles" in brain neurons of a patient with presenile dementia.^1^ A short time later this condition received the denomination of "Alzheimer's disease".^2^

Alzheimer's interests were quite wide, including vascular brain diseases. His precursor work greatly contributed toward introducing key knowledge of what would later be incorporated into the field of "Vascular dementia" and related disorders. Although he was not the only scholar to study this subject, he produced very comprehensive concepts, including novel pathological descriptions on vascular brain diseases.^3^

At the time, the dementia definition was somewhat loose, and comprised heterogeneous mental conditions, including General paralysis, melancholia, mania, Senile dementia, varied psychic disorders of the senium, vascular brain diseases, and others.^2,4,5^ Alzheimer and numerous famous scientific contemporaries focused on extricating these many disorders according to their etiology, and mainly on differentiating them from the prevalent syphilitic brain disorders.^6,7^ This body of work resulted in advances to a field in which knowledge had remained stagnated since Antiquity.^4,8,9^

The objective of the present paper was to discuss Alzheimer's studies on Senile dementia, a condition to be later subdivided into several independent diseases.

## SENILE DEMENTIA

Alzheimer previously mentioned the term Senile dementia (*Dementia senilis, senile Demenz*) in the main text and discussion of his 1894 paper.^10^ He further commented, in 1898, about the lack of systematic clinicopathological studies on Senile dementia, yet an abundance of studies on Paralytic dementia (Paralysis).^6^ Additionally, he highlighted that most studies on Senile dementia had included conditions that more closely resembled mental disorders of the senium or senile mental impairment (*Greisenblödsinn*) such as melancholia, mania, depression, among others, which might equally be related to arteriosclerosis, as cited by Kraepelin. Such disorders of the senium could eventually show regressive changes, with feebleness and reduced brain performance, finally evolving to Senile dementia.^2,6^ Thus, regarding this conceptual heterogeneity, Alzheimer stated that "Senile dementia may develop in the form of extraordinary colorful and richly varied pictures, making it difficult to classify the disease based solely on clinical symptoms, similarly to the challenges in Paralysis".^6^

Alzheimer discussed the possible underlying pathology of Senile dementia and the correlation with clinical manifestations He cited Weigert's work that demonstrated an elevated number of glia in the upper cortical layers [astrogliosis], and that their processes constituted a dense fiber meshwork in the cerebral grey and white matter. He further suggested a correlation between astrogliosis severity and cognitive decline, highlighting that older brains with milder glial changes may manifest less severe regression of mental performance.^6^ However, these changes often reached higher degrees of severity and caused the milder or "silent form" of Senile dementia (*stille Form der Dementia senilis*), possibly the most frequent mental disorder, found commonly among patients living with their families or in asylums, and rarely in institutions, as also observed by Windscheid.^11-13^ Other cases, however, exhibited a progressive course, finally reaching a marked degree of dementia, with serious manifestations of senile mental impairment (*Alterblödsinn*).^6^

In the same paper, Alzheimer cited Noetzli, who following Forel's opinion, expressed that the silent form of Senile dementia might affect everyone, possibly more so those predisposed to atheromatous disease, whereas individuals with more advanced manifestations accompanying age related-mental impairment required a psychic hereditary load, in agreement with some authors that held the opinion that a 2^nd^ item must be added to elucidate the process.^14^ This view, despite its tempting appeal, seemed barely demonstrable to Alzheimer.^6^ Later, Noetzli himself referred to atheromatous degeneration as the fundamental cause of Senile dementia, contradicting his former belief and in agreement with Alzheimer's previous opinion.^6^ Thus, at the time, most researchers agreed that atheromatous degeneration of the brain vessels was the underlying mechanism of Senile dementia.

Alzheimer, however, reconsidered this position after examining a case classified as presenile dementia featuring severe atrophy of [cortical] ganglion cells [nerve cells, neurons], but with negligible atheromatous vessel changes. The case, at least for this form, contradicted Noetzli's latest views, and prompted Alzheimer to consider the possibility that a hereditary load might lead to precocious atrophy of the nerve cells independently of vessel disease. These results encouraged him to propose that degenerative changes of the nerve cells might also appear, independently of a vascular disease, in cases of typical Senile dementia. Therefore, an influence of psychic hereditary load on the cause of Senile dementia would make more sense. However, he concluded that a single case could not be considered final proof, and further investigation was needed to support this assumption.^6,11^

It is pertinent to summarize Jean Noetzli's and Alfred Campbell's data on brain weight, and Campbell pathological analysis for a better understanding of brain pathology of insane patients. Noetzli, Forel's adherent, in his inaugural dissertation *Ueber Dementia Senilis* (On Senile dementia),^14^ described a clinicopathological analysis of 70 cases mostly dissected by Forel himself. He provided data on the brain weight of 40 selected cases of "senile psychosis without focal symptoms", which displayed a significant loss of brain weight [brain atrophy] in both genders, compared to controls.^6,14^ Along the same lines, Campbell detected brain atrophy among males and females, in a comprehensive study of the nervous system of 50 patients that he called "aged insane".^15^ Campbell also provided detailed gross and microscopic description of the material. Alzheimer apparently considered "aged insane" and Senile dementia equivalent, transcribing part of Campbell's text in his 1898 paper^6^ (Box 1).

**Box 1:** Excerpts on the neuropathology of Senile dementia, based on Campbell’s paper.^6,15^

The pathological aspects of Senile dementia were mostly based on the detailed examination of the histologic changes reported by Campbell’s paper on the neuropathology of “aged insane”, a term that Alzheimer regarded as a comparable condition.
The superficial layers of the cerebral cortex appeared densely fibrillated, and the surface of the cerebral cortex showed abundant *Corpora* *amylacea*, especially numerous in the external medullary lamina of Ammon’s horn. He also described the occurrence of numerous spider cells (*Spinnenzellen, astrocytes*) in the superior layers, viewed as an almost characteristic sign of Senile dementia. He described small and evenly scattered gold yellowish pigment in the body of these cells or in their processes. Campbell believed they were almost a characteristic feature of “senile insanity” [Senile dementia] that could be differentiated from those found in Paralysis.
A widespread degeneration of the cortical neurons, in all stages of breakdown, could be observed. The cells showed typically a pigmentary degeneration, which affected nerve cells of all sizes, in which protoplasm was entirely replaced by the pigment, and amorphous clusters dispersed in the tissue (matrix) showed the final remains of the pigmentary degeneration, indicating the position of a former nerve cell, now disintegrated (*).
The constituents of the cortical blood vessels could not be clearly distinguished. The microscopic changes were distinctive, with marked *arteritis chronica deformans*, embolic, and thrombotic softenings, perivascular hemorrhages, with destruction of the surrounding tissue in the basal nuclei and in the pons Varolii, common changes in morbid senescence. The perivascular spaces were mostly dilated, particularly in the sections through the basal ganglia (*état criblé*, according Durand-Fardel, 1854).
The macroscopic changes of the brain comprised atrophy and decrease in weight, thinned convolutions, wide, shallow, open sulci. The frontal segment invariably suffered most change. The surface of the cerebrum was generally firm, sometimes slightly puckered. On sectioning, the cortex appeared shallow, dark in color, with indistinct striation, and the white matter was atrophied in proportion to the surface atrophy. The ventricles were always greatly expanded with an excess of fluid (“*hydrocephalus ex vacuo*”).
(*) Concerning this microscopic description. it should be noted that at the time, specific silver staining techniques were not yet available, therefore the description must be interpreted with caution.

Alzheimer also acknowledged that: "Besides the typical Senile dementia, where diffuse changes in cerebral cortex are found, it was still possible to distinguish among the senile psychosis, various disease forms (subforms), well-characterized clinically and histologically, having arteriosclerotic degeneration of brain vessels in common as the underlying cause, and equally observed in the aged. Many of these subforms, depending on the base disease, can resemble the clinical picture of Senile dementia". He affirmed that some of these conditions are to be considered as mere subforms of *Arteriosklerotische Hirnatrophie* (Arteriosclerotic atrophy of the brain).^6,16^ The subforms he mentioned will be addressed later.

Concluding, Alzheimer stated that: "Regarding Senile dementia, it is necessary to see with reservation whether arteriosclerosis of the brain vessels should be considered as the only underlying cause of senile degeneration of the brain, or whether primary atrophic processes of ganglion cells should also be taken into consideration. In the remaining disease forms, atheromatous vessel degeneration is clearly a central factor in the degenerative process."^6,11^

## COMMENTARIES

Based on his own observations, in addition to work by Weigert, Noetzli and Campbell , Alzheimer was a pioneer in conceptualizing Senile dementia as a disorder featuring diffuse changes of the cerebral cortex and subcortical structures, and distinct from syphilitic disease, one of his objectives.^6,15^

Alzheimer extricated from the group of "senile psychosis", many of them at the time included under the umbrella-label of Senile dementia, two subtypes of disorders,^6,11^ which can be denoted as an "arteriosclerotic subtype" (Box 2) and a "neurodegenerative subtype" (Box 3).

**Box 2:** The “arteriosclerotic subtype” of Senile dementia.

This subtype refers, as understood by the present authors, to brain degeneration due to arteriosclerotic load of the nervous structures, as atheromatous degeneration was deemed the underlying mechanism of Senile dementia by many researchers at the time (e.g. Noetzli, Campbell, Alzheimer, Klippel, and Marcé)^6,12,17^.
It is opportune to comment that, comparing the “arteriosclerotic subtype” of Senile dementia with Arteriosclerotic atrophy of the brain^3^, considerable clinicopathological overlap may be seen, making it difficult, in some instances, to differentiate between them.
Reinforcing this line of reasoning, it is important to remember the discussion held in his 1884 paper on Arteriosclerotic atrophy of the brain among Binswanger, Moeli, Sioli, Kraepelin, Jolly, and Alzheimer. Among those that commented specifically on the issue, Moeli, Jolly, and the author held conflicting opinions on the view that the condition in question was to be considered similar to Senile dementia^10^. Additionally, in his 1898 paper, Alzheimer stated: “Besides the typical Senile dementia, in recent years, more varied clinical and histological well characterized disease pictures have been better identified in which underlying atheromatous vessel degeneration was responsible for the disease process, and that are more frequently observed in the aged”^6^. And in the following year he wrote: “Besides the Senile dementia, where diffuse changes in the cerebral cortex were found, it is possible to identify among the senile psychosis varied focal disease, in which arteriosclerosis of the brain vessels had to be considered as a common cause”. And completing the paragraph, he stated: “All these forms however, are only to be regarded as subforms of Arteriosclerotic atrophy of the brain”^16^.
Thus, taking into account all of the above considerations, a clear overlap can be seen between both conditions, which may suggest that these two presentations refer to a single entity.

**Box 3:** The “neurodegenerative subtype” of Senile dementia

The “neurodegenerative subtype”, as understood by the present authors, was based on severe primary atrophy of [cortical] ganglion cells [nerve cells, neurons], with insignificant atheromatous vascular changes. It is possible that this subtype foreshadowed the disease he later described, regarding another presenile demented patient he encountered in 1901 (Auguste D.) and whose brain he studied in 1906. The Bielschowsky’s silver staining technique that appeared in 1903 permitted identification of remarkable “neurofibrillary changes” (*sehr merkwürdige Veränderungen der Neurofibrillen*), and additionally, “miliary nodules” (*miliare Herdchen*), mostly abundant in the superficial layers. This “new disease”^1^ was later designated as “Alzheimer’s disease”^2,20^.
Alzheimer also assumed that “…in cases of typical Senile dementia, degenerative changes of the ganglion cells might appear, independent of a vascular disease”^6^. This supposition was soon confirmed by Fischer’s studies on Presbyophrenic dementia (*Die presbyophrene* *Demenz*) (from Presbyophrenie, according to Wernicke)^22^, a clinical form of Senile dementia (*senile Verblödung*), according to Kraepelin^2^. In 1907, Fischer described plaques (drusigen Nekrosen, Sphaerotrichia cerebri multiplex)^21^, apparently the main focus of his study at the time. In 1910, he went on to describe neurons with “neurofibrillary changes” (“*grobfaserige Fibrillenwucherung der Ganglienzellen*”) in a number of senile cases^22^.
Thus, plaques (*miliare Herdchen, drusigen Nekrosen*) and neurofibrillary changes (*sehr merkwürdige Veränderungen der Neurofibrillen*, *grobfaserige Fibrillenwucherung der Ganglienzellen*), found in presenile and senile cases, became a hallmark of the neurodegenerative subtype of the disease.

Additionally, Alzheimer, as did Weigert, Noetzli, and Windscheid, provided insights on the relationship between neuropathological and clinical severity. Milder pathology was associated with lesser cognitive decline. However, he added, the disease could worsen leading to the mild or "silent form" of Senile dementia and even progress to a severe dementia state, accompanied eventually by several kinds of behavioral disturbances.^6^

The "arteriosclerotic subtype" of Senile dementia (Box 2) and the several grades of clinicopathological presentations he described, also quoting other colleagues, might be considered comparable to the current dimensional concept of the "Vascular cognitive impairment" continuum.^18^ It is opportune to reiterate that this was verified and suggested in relation to Arteriosclerotic atrophy of the brain in a previous paper.^3^ An analyzes of Senile dementia based on Alzheimer's 1898 paper was performed by Förstl and Howard, where they commented on the clinical progression of Senile dementia etiopathogenesis according to Noetzli and Alzheimer, and on Arteriosclerotic brain atrophy.^19^ In 1999, Román commented on Senile dementia, focusing on the main points of Alzheimer's study, and made remarks on Arteriosclerotic brain atrophy and also on some subforms.^7^

The "neurodegenerative subtype" of Senile dementia (Box 3) was based upon severe primary atrophy of [cortical] ganglion cells [nerve cells, neurons], with insignificant atheromatous vascular changes, found in the material of a presenile dementia case.^6,20^ He extended this possibility to senile cases, an issue confirmed later by Oscar Fischer's studies.^22^ Román,^7^ as well as Derouesné,^17^ also commented on this possibility.

## CONCLUSION

Alzheimer was able to distinguish Senile dementia from syphilitic brain disease. Additionally, he discriminated two clinicopathological subtypes, an "arteriosclerotic subtype", possibly related to "Vascular cognitive impairment", and a "neurodegenerative subtype", characterized by primary [cortical] nerve cell degeneration, in a presenile dementia case, possibly foreshadowing a disease he described years later that would carry his name, "Alzheimer' disease". He extended the latter possibility to also encompass senile cases, confirmed later by Fischer's studies.
